# Activation of TFEB ameliorates dedifferentiation of arterial smooth muscle cells and neointima formation in mice with high-fat diet

**DOI:** 10.1038/s41419-019-1931-4

**Published:** 2019-09-12

**Authors:** Yun-Ting Wang, Xiang Li, Jiajie Chen, Bradley K. McConnell, Li Chen, Pin-Lan Li, Yang Chen, Yang Zhang

**Affiliations:** 1 School of Pharmaceutical, Guangzhou University of Chinese Medicine, Guangzhou, China; 2 Department of Pharmacological and Pharmaceutical Sciences, College of Pharmacy, University of Houston, Houston, TX USA; 3 Department of Biology and Biochemistry, University of Houston, Houston, TX USA; 4 Department of Pharmacology and Toxicology, School of Medicine, Virginia Commonwealth University, Richmond, VA USA

**Keywords:** Carotid artery disease, Carotid artery disease

## Abstract

Autophagy is recently implicated in regulating vascular smooth muscle cell (SMC) homeostasis and in the pathogenesis of vascular remodeling. Transcription factor EB (TFEB) is a master regulator of autophagy signaling pathways. However, the molecular mechanisms and functional roles of TFEB in SMC homeostasis have not been elucidated. Here, we surveyed the ability of TFEB to regulate autophagy pathway in SMCs, and whether pharmacological activation of TFEB favors SMC homeostasis preventing dedifferentiation and pathogenic vascular remodeling. In primary cultured SMCs, TFEB activator trehalose induced nuclear translocation of TFEB and upregulation of TFEB-controlled autophagy genes leading to enhanced autophagy signaling. Moreover, trehalose suppressed serum-induced SMC dedifferentiation to synthetic phenotypes as characterized by inhibited proliferation and migration. These effects of trehalose were mimicked by ectopic upregulation of TFEB and inhibited by TFEB gene silencing. In animal experiments, partial ligation of carotid arteries induced downregulation of TFEB pathway in the media layer of these arteries. Such TFEB suppression was correlated with increased SMC dedifferentiation and aggravated high-fat diet (HFD)-induced neointima formation. Treatment of mice with trehalose reversed this TFEB pathway suppression, and prevented SMC dedifferentiation and HFD-induced neointima formation. In conclusion, our findings have identified TFEB as a novel positive regulator for autophagy pathway and cellular homeostasis in SMCs. Our data suggest that suppression of TFEB may be an initiating mechanism that promotes SMC dedifferentiation leading to accelerated neointima formation in vascular disorders associated with metabolic stress, whereas trehalose reverses these changes. These findings warrant further evaluation of trehalose in the clinical settings.

## Introduction

Vascular smooth muscle cells (SMCs) is a major cell type within the media layer of the arterial wall. SMCs within the adult blood vessel are maintained in homeostatic status and possess contractile phenotype with very low synthetic activity. Under pathogenic conditions, SMCs can be activated and dedifferentiated to a synthetic state that SMCs become proliferative and migratory leading to intimal hyperplasia and luminal stenosis^1,2^. The deregulation of SMC phenotypic plasticity due to impaired SMC homeostasis is a pathogenic basis of several different vascular disorders including atherosclerosis^3^, postangioplasty restenosis^4^, and vein graft stenosis^5^. Metabolic stress associated with obesity and dyslipidemia is a major cardiovascular risk factor for the development of vasculopathy. Many factors associated metabolic stress can modulate SMC homeostasis including various adipokines such as adiponectin and lipid mediators such as free fatty acids. Therefore, there are great clinical implications in understanding the mechanisms underlying the dynamic regulation of SMC homeostasis or phenotypic plasticity under metabolic stress and establishing related pharmacological interventions.

Autophagy is an evolutionarily conserved, nonstop, reparative, and life-sustaining catabolic process to maintain normal cellular homeostasis. In this process, double-membraned autophagosomes engulf unhealthy organelles and long-lived proteins and degrade them into small molecules via autophagic flux. In autophagic flux, autophagosomes fuse with acidic lysosomes to form autophagolysosomes, in which autophagic substrates are degraded by lysosome proteinases and hydrolyases^6–9^. Accumulating evidence indicate that moderately enhanced autophagy in the vasculature protects against progressive atherosclerosis or restenosis, whereas defective or excessive autophagy is promoting these disease states^10^. In particular, autophagy plays a critical role in maintaining SMC homeostasis or regulating its phenotypic plasticity^11,12^. Increased autophagy blunts proliferation in SMCs under various atherogenic stimuli such as thrombin and advanced glycation end products^13–16^. Enhanced but not excessive autophagy by either statins or rapamycin helps maintain vascular smooth muscle in contractile phenotype and inhibits their proliferation^15,17^. Moreover, augmented autophagy was found to inhibit SMC migration^18,19^. On the contrary, defective autophagy due to impaired autophagy induction contributes to uncontrolled cell growth or migration^20–22^, which promotes neointimal lesions in the arterial wall as well as atherosclerotic development^22–24^. In addition to impaired autophagy induction, the defective autophagy pathway is also associated or caused by lysosome dysfunction or injury^7,25^. In mice with genetic deletion of LAMP2, a lysosome associated membrane protein that is critical for lysosome function, aberrant autophagy was observed in SMCs that was linked to luminal stenosis and medial thickening^26^. Our recent studies also demonstrated that impaired autophagy maturation due to lysosome dysfunction contributes to imbalanced SMC homeostasis^7,25,27^. In this study, we speculate if enhancement of both autophagy induction and lysosome biogenesis pathways in SMCs comprises a promising strategy that may efficiently reduce aberrant proliferation and migration of SMCs, and eventually prevent intimal hyperplasia and luminal stenosis.

Transcription factor EB (TFEB) is a member of the microphthalmia family of basic helix-loop-helix–leucine-zipper (bHLH-Zip) transcription factors (MiT family)^28–30^. Recent studies highlight TFEB as a master controller of autophagy pathway by driving the expression of autophagy and lysosomal genes^30–36^. Once activated, TFEB translocates into nuclei and binds to coordinated lysosomal expression and regulation elements of target genes and thereby promoting their expression^31,37–39^. Activation of TFEB induces autophagosome formation and lysosome biogenesis, both of which contributes to enhanced cellular clearance capability via autophagy^28,30,31,40^. The role of TFEB in regulation of cardiovascular functions has only recently become clearer. In particular, activation of TFEB by trehalose ameliorates atherosclerosis development in mice by promoting lysosome regeneration, autophagy induction, and inhibition of inflammasome activity in macrophages^41–43^. Further, endothelial specific overexpression of TFEB suppresses endothelial inflammation and attenuate atherosclerosis in mice^44^. However, the functional roles of TFEB in SMCs and vascular remodeling have not been investigated.

Trehalose is a nonreducing disaccharide composed of two d-glucose units linked α-1,1. Trehalose is synthesized by some bacteria, fungi, certain plants, and invertebrate animals as an energy source to survive freezing and water-deficit environments^45^. Trehalose is commonly used as a stabilizer excipient in numerous medicines in pharmaceutical industry, and a sweetener in the nutraceutical industry^46^. Recently, trehalose was identified as a potent TFEB activator that induces autophagy signaling in a variety of mammalian cells^42,47^. Moreover, trehalose was shown to exhibit protective effects on atherosclerosis, which is attributed to enhanced TFEB-dependent autophagy signaling in macrophages^42,43^. This study aims to determine whether trehalose treatment can restore autophagy signaling pathway in SMCs and thereby correct the impaired SMC homeostasis, which in turn contributes to prevention of neointimal injury induced by high-fat diet (HFD). In this respective, we first sought to examine the role of TFEB in autophagy signaling in cultured SMCs and their proliferation and migration by using trehalose treatment and TFEB gain-of-function studies. In animal experiments, we also investigated whether suppression of TFEB-mediated signaling is correlated with SMC activation and dedifferentiation in the arterial wall and whether trehalose can reverse this TFEB suppression and SMC dedifferentiation contributing to prevention of HFD-induced neointimal formation. These findings provide new insights into the pathogenesis of vasculopathy associated with metabolic stress.

## Results

### Trehalose activates TFEB-mediated autophagy signaling in cultured SMCs

**Fig. 1:**
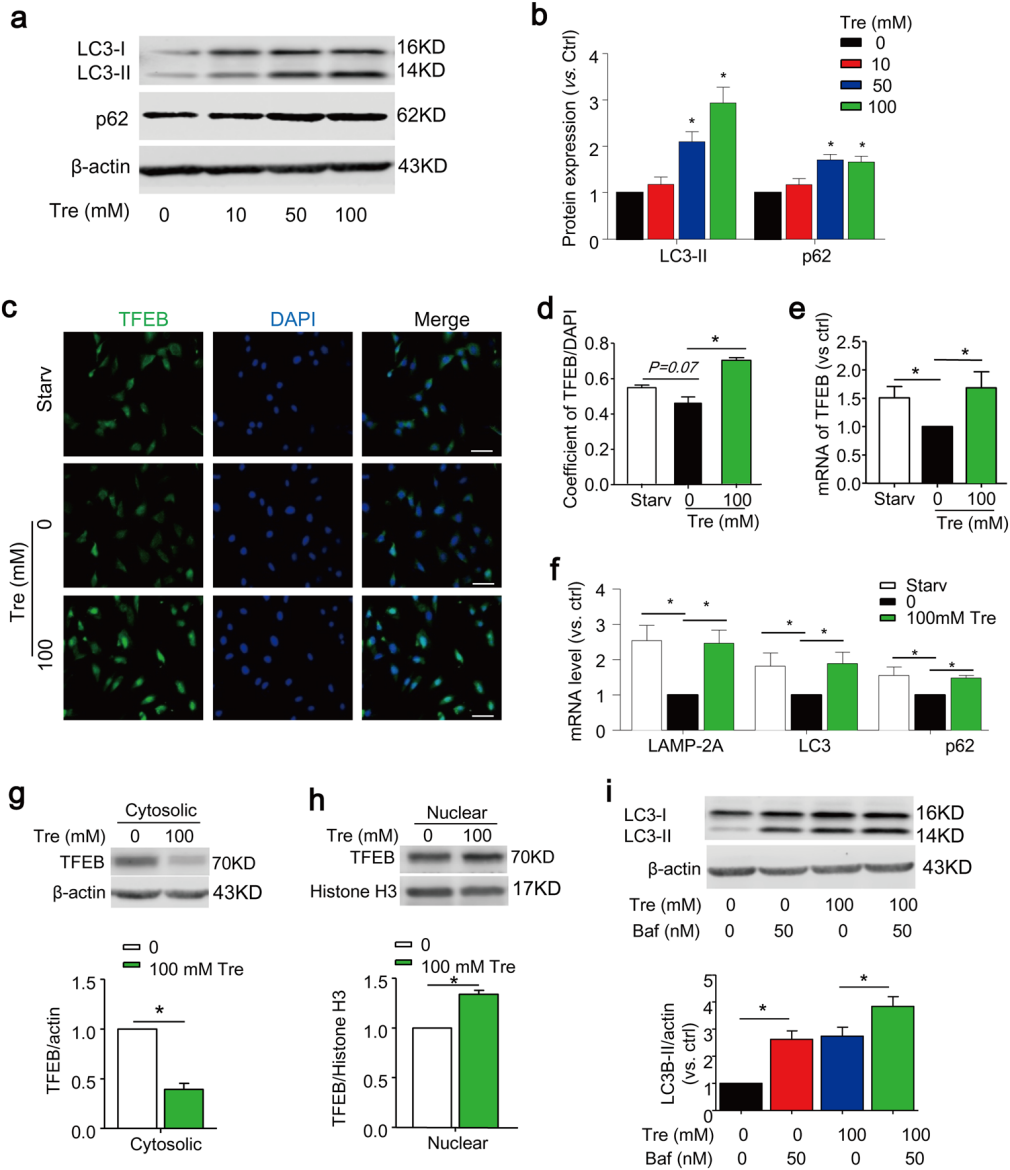
Trehalose activates TFEB-mediated autophagy signaling in cultured SMCs. **a**, **b** SMCs were cultured in full-serum medium (10% FBS) and treated with trehalose (0–100 mM) for 24 h. Representative immunoblots and summarized data show the effects of trehalose on the protein expression levels of LC3-II and p62/SQSTM1. **c**–**f** SMCs treated with or without 100 mM trehalose cultured in full-serum medium or under starvation condition (Starv, 0.1%FBS) for 24 h. Representative immunofluorescence images (**c**) and quantification data (**d**) show the nuclear translocation of TFEB (green). Nuclei were stained with DAPI. **e**, **f** Real-time RT-PCR analyses of TFEB, LAMP-2A, LC3 and p62/SQSTM1 mRNA levels. **g**, **h** Immunoblots show the expression of TFEB in cytosolic or nuclear extract of SMCs (*n* = 4). **i** SMCs cultured in full-serum medium were treated with or without 100 mM trehalose in the absence or presence of bafilomycin (Baf, 50 nM) for 24 h. Representative immunoblots and summarized data show the protein expression levels of LC3-II. Scale bar = 50 µm. *P < 0.05 (*n* = 4)

Trehalose is a nonreducing disaccharide composed of two d-glucose units linked α-1,1 and recently it was reported to induce autophagy via activation of TFEB^42,47^. We first sought out to examine whether TFEB activator trehalose could affect autophagy signaling in SMCs cultured in full-serum media (10% FBS). As shown in Fig. 1a, b, trehalose significantly increased the protein expression of autophagy marker LC3-II and autophagic substrate p62/SQSTM1. TFEB is a master transcription factor of genes involved in autophagy induction and lysosome biogenesis as well as TFEB itself. Indeed, trehalose-induced changes in protein expression of autophagy and lysosome genes were accompanied by increased nuclear translocation of TFEB (Fig. 1c, d), induction of mRNA transcription of TFEB gene (Fig. 1e) and downstream genes including autophagy markers LC3, p62/SQSTM1, and lysosome markers LAMP-2A (Fig. 1f). Serum starvation in culture media with 0.1% FBS was shown to increase TFEB activity and autophagy signaling in endothelial cells^48^. Consistent to that in endothelial cells, we observed that SMCs under serum starvation condition had increased nuclear translocation of TFEB (*P* = 0.07) and higher mRNA levels of TFEB and its downstream genes (LC3, p62/SQSTM1, LAMP-2A) compared with normal controls (Fig. 1c–f). The nuclear translocation by trehalose was also confirmed by a decrease of TFEB expression in the cytosol (Fig. 1g) and a corresponding increase in its nuclear expression (Fig. 1h). When autophagic flux was blocked by bafilomycin, trehalose further increased LC3 expression indicating that trehalose-induced increase in autophagy markers is not associated with impaired lysosome clearance (Fig. 1i). Taken together, these data indicate that TFEB signaling pathway is present in SMCs and can be activated by trehalose or under serum starvation condition.

### Activation of TFEB by trehalose inhibits proliferation in cultured SMCs

**Fig. 2:**
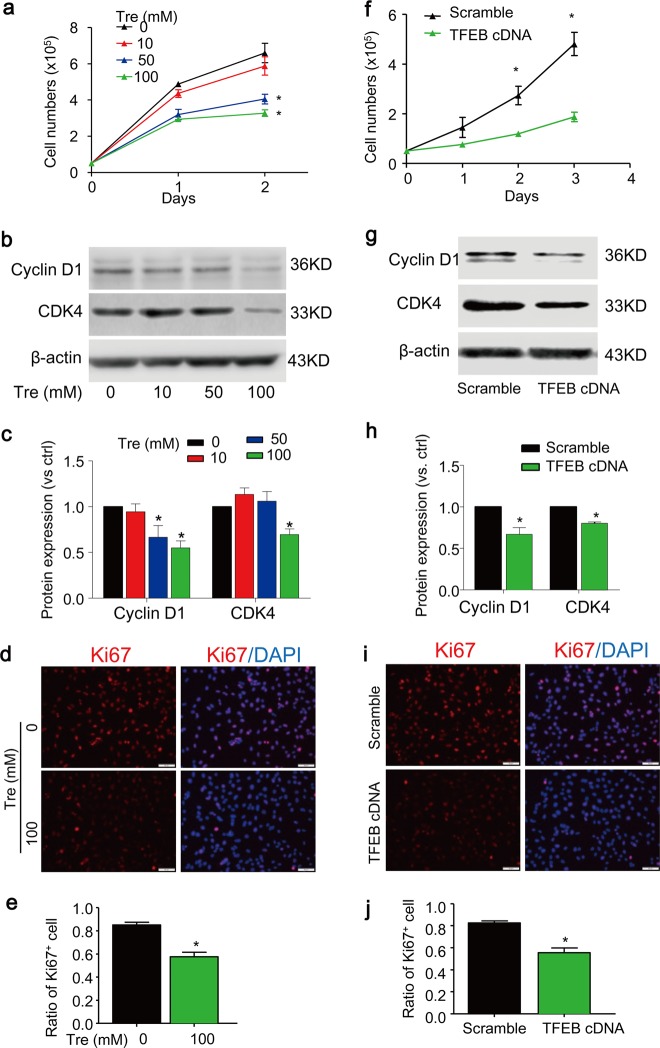
Trehalose inhibits proliferation of SMCs. **a**–**e** SMCs cultured in full-serum medium were treated with trehalose (0–100 mM) for indicated time points. Cell proliferation was analyzed by counting the cell numbers (**a**). Immunoblotting analysis (**b**, **c**) shows the effects of trehalose (24 h) on the cell cycle protein cyclin D1 and CDK4. **d**–**e** Immunofluorescence images show the proliferative cell marker Ki67 (red) in SMCs treated with vehicle or 100 mM trehalose for 24 h. Nuclei were stained with DAPI. **f–j** SMCs were transfected with scramble or TFEB cDNA plasmids for 24 h and then cultured in full-serum medium for indicated time points. **f** Cell number counting. **g**, **h** Immunoblotting analysis shows the effects of TFEB overexpression on cyclin D1 and CDK4 in SMCs 48 h after transfection. **i**, **j** Immunofluorescence images for Ki67 (red) in SMCs transfected SMC SMCs 48 h after transfection. Scale bar = 50 µm *P < 0.05 (*n* = 4)

As shown in Fig. 2a, a significant decrease in cell growth was observed in primary cultured SMCs when they were incubated with trehalose at a concentration higher than 50 mM. Consistently, trehalose significantly decreased the expression of cell cycle proteins cyclin D1 and CDK4 (Fig. 2b, c) and proliferative cell marker Ki67 (Fig. 2d, e). Moreover, trehalose did not increase cell death in cultured SMCs in the same experimental settings (supplementary Fig. S1).

We also confirmed the role of TFEB in proliferation by TFEB gain-of-function studies. The transfection efficiency of TFEB cDNA was examined by western blot analyses (supplementary Fig. S2). TFEB-overexpressed SMCs showed reduced proliferation rate as determined by growth curve (Fig. 2f). Consistently, TFEB-overexpressed cells have an impaired cell cycle as shown by reduced expression of cell cycle related proteins cyclin D1 and CDK4 (Fig. 2g, h). The anti-proliferative effects of TFEB overexpression were also confirmed by decreased expression of proliferation marker Ki67 **(**Fig. 2i, j**)**. Together, these data suggest that activation of TFEB by trehalose exerts anti-proliferative effects in SMCs, which is not associated with its cytotoxic effects.

### Activation of TFEB by trehalose inhibits migration of SMCs in vitro

**Fig. 3:**
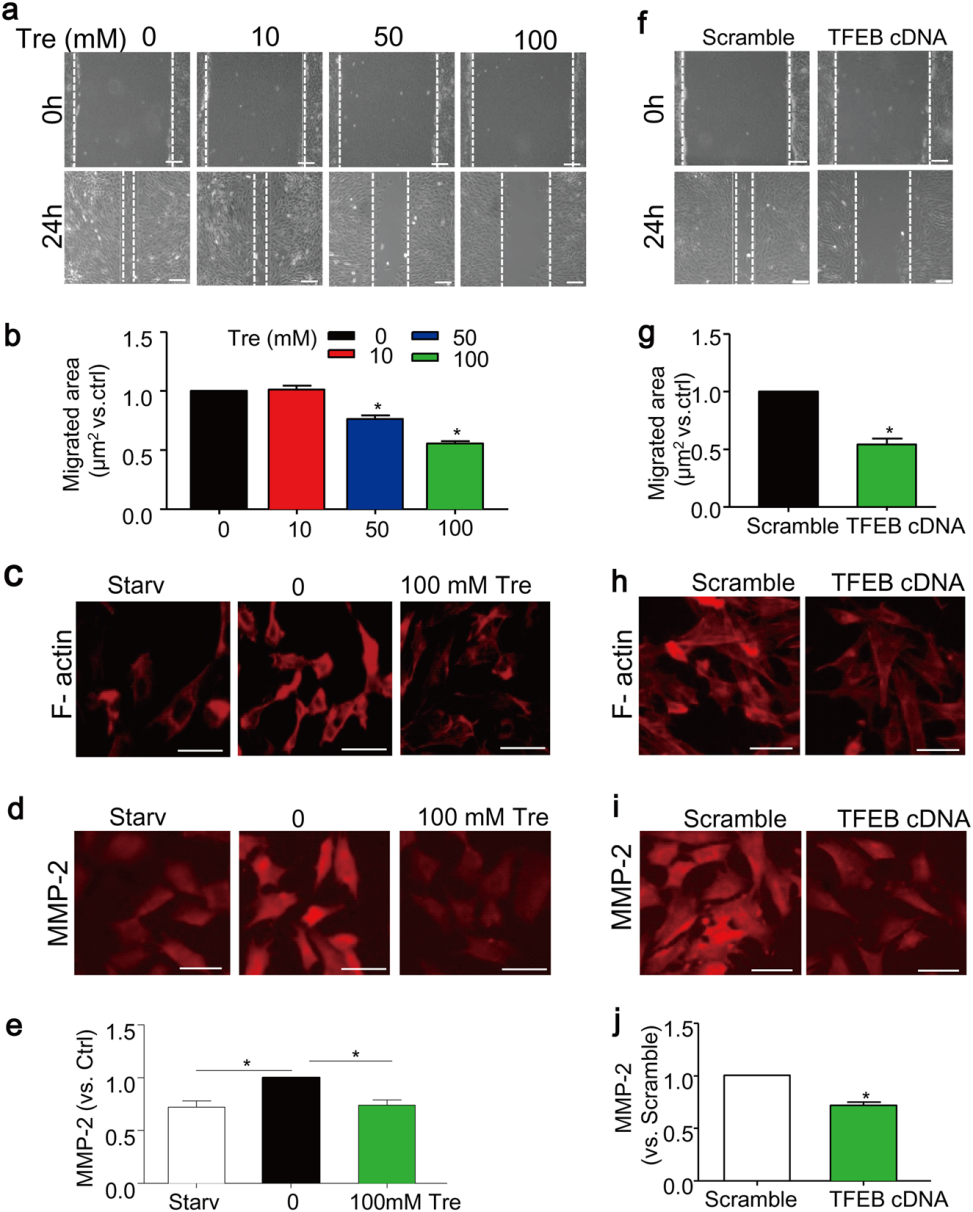
Trehalose inhibits migration of SMCs. **a**, **b** SMCs cultured in full-serum medium were treated with trehalose (0–100 mM) for 24 h. Migration was analyzed by scratch assay. **c**–**e** SMCs were treated with vehicle or 100 mM trehalose cultured in full-serum medium, or under starvation condition (Starv, 0.1%FBS) for 24 h. Immunofluorescence images (**c**) show the arrangement of F-actin filaments by phalloidin staining (red). **d**, **e** Immunofluorescence analysis of MMP2 expression in SMCs. **f–j** SMCs were also transfected with scramble or TFEB cDNA plasmids for 24 h and then cultured in full-serum medium for another 24 h. **f**, **g** Scratch assay of transfected SMCs. **h** Immunofluorescence images show the F-actin filaments of transfected SMCs. **i**, **j** Immunofluorescence analysis of MMP2 expression in transfected SMCs. Scale bar = 50 µm *P < 0.05 (*n* = 4)

As shown in Fig. 3a, b, trehalose treatment significantly decreased migration of SMCs as assessed by the scratch assay. The remodeling of filamentous actin cytoskeleton (F-actin) is known to be a marker event associated with cell migration^49^. Here, we found that SMCs cultured in control condition (full-serum media with 10% FBS) exhibited migrative phenotype with disassembled distribution and aggregation around the perinuclear region of actin filaments without clear filamentous organization (Fig. 3c). In contrast, cells treated with trehalose maintained spindle-like shapes and organization of the actin filaments, which resembles the phenotype in SMCs under serum starvation condition (0.1% FBS), a condition known to induce differentiation of SMCs and inhibit their migration (Fig. 3c). Trehalose also decreased the expression of matrix metalloproteinases-2 (MMP2) **(**Fig. 3d, e**)**, which is a peptidase enzyme involved in extracellular matrix degradation^50^. Similar to trehalose, overexpression of TFEB significantly decreased the migration of SMCs (Fig. 3f, g), induced F-actin re-organization **(**Fig. 3h), and downregulated MMP-2 expression **(**Fig. 3i, j**)**.

### TFEB gene silencing attenuates the effects of trehalose on SMC proliferation and migration

**Fig. 4:**
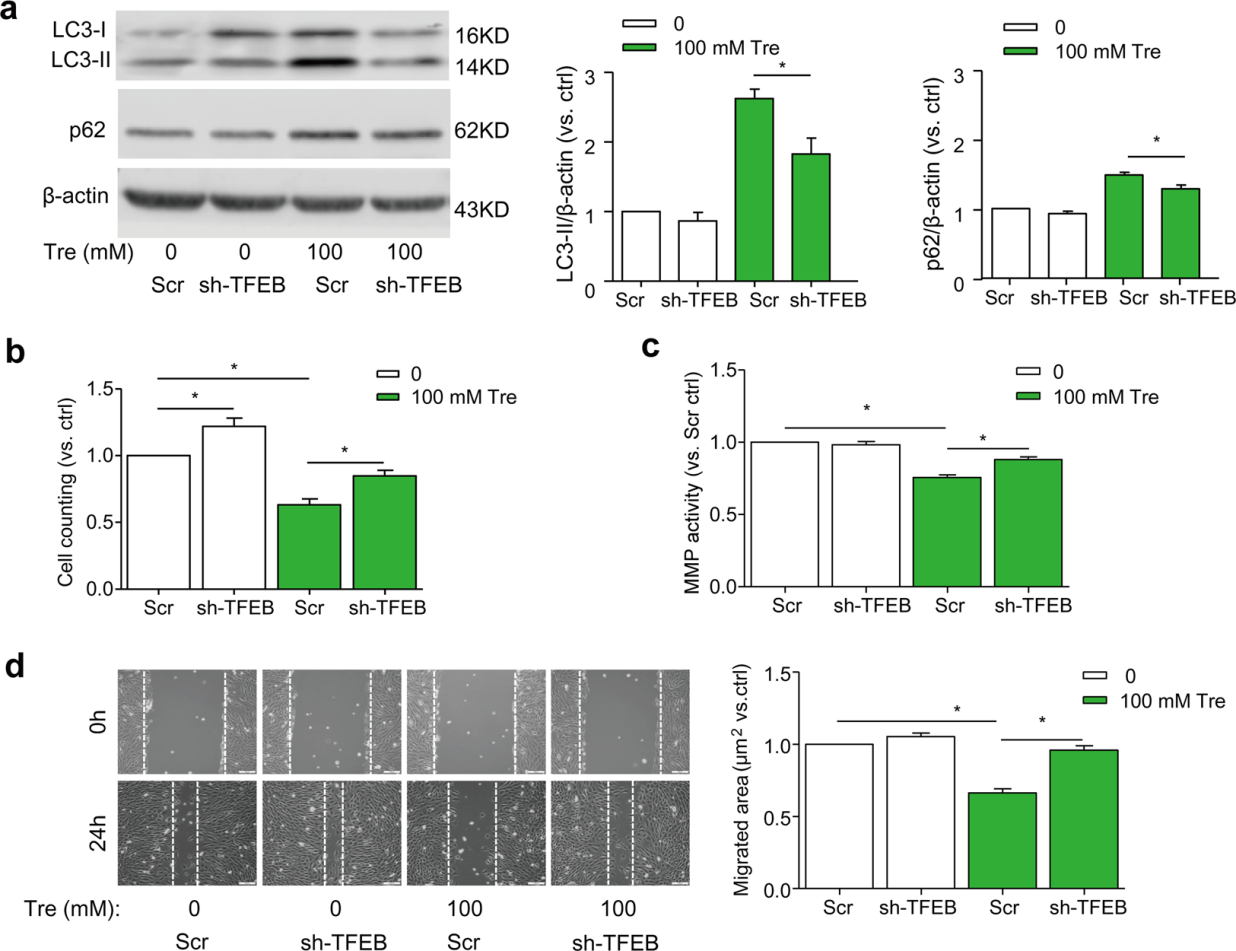
TFEB gene silencing ameliorates trehalose-induced proliferation and migration of SMCs. SMCs in full-serum medium were transduced with scramble or TFEB shRNA lentiviral particles as described in “Methods”. The cells were treated with vehicle or trehalose (100 mM). **a** Immunoblot analysis show the effects of TFEB silencing on protein expression of LC3 and p62/SQSTM1 in SMCs with trehalose for 24 h (*n* = 6). **b** Cell number counting after 24-h trehalose (*n* = 6). **c** MMP activity of SMCs after 48-h trehalose by a fluorometric MMP activity assay kit. (*n* = 4). **d** Scratch assay of SMCs with trehalose for 24 h (*n* = 6). Scale bar = 100 μm *P < 0.05 vs Scr

TFEB gene silencing by TFEB shRNA transfection effectively decreased expression of TFEB in SMCs (Supplementary Fig. S3) and significantly attenuated trehalose-induced upregulation of LC3 and p62 (Fig. 4a). When the TFEB gene is silenced in SMCs, trehalose-induced inhibition of proliferation was significantly attenuated (Fig. 4b). Moreover, trehalose-induced inhibition of MMP activity (Fig. 4c) and SMC migration (Fig. 4d) were reversed by TFEB gene silencing. Together, these data suggest that trehalose treatment reduces the proliferative and migratory capacity of SMCs, which is mediated through TFEB pathway.

### TFEB-mediated autophagy signaling is defective in the arterial media of PLCAs

**Fig. 5:**
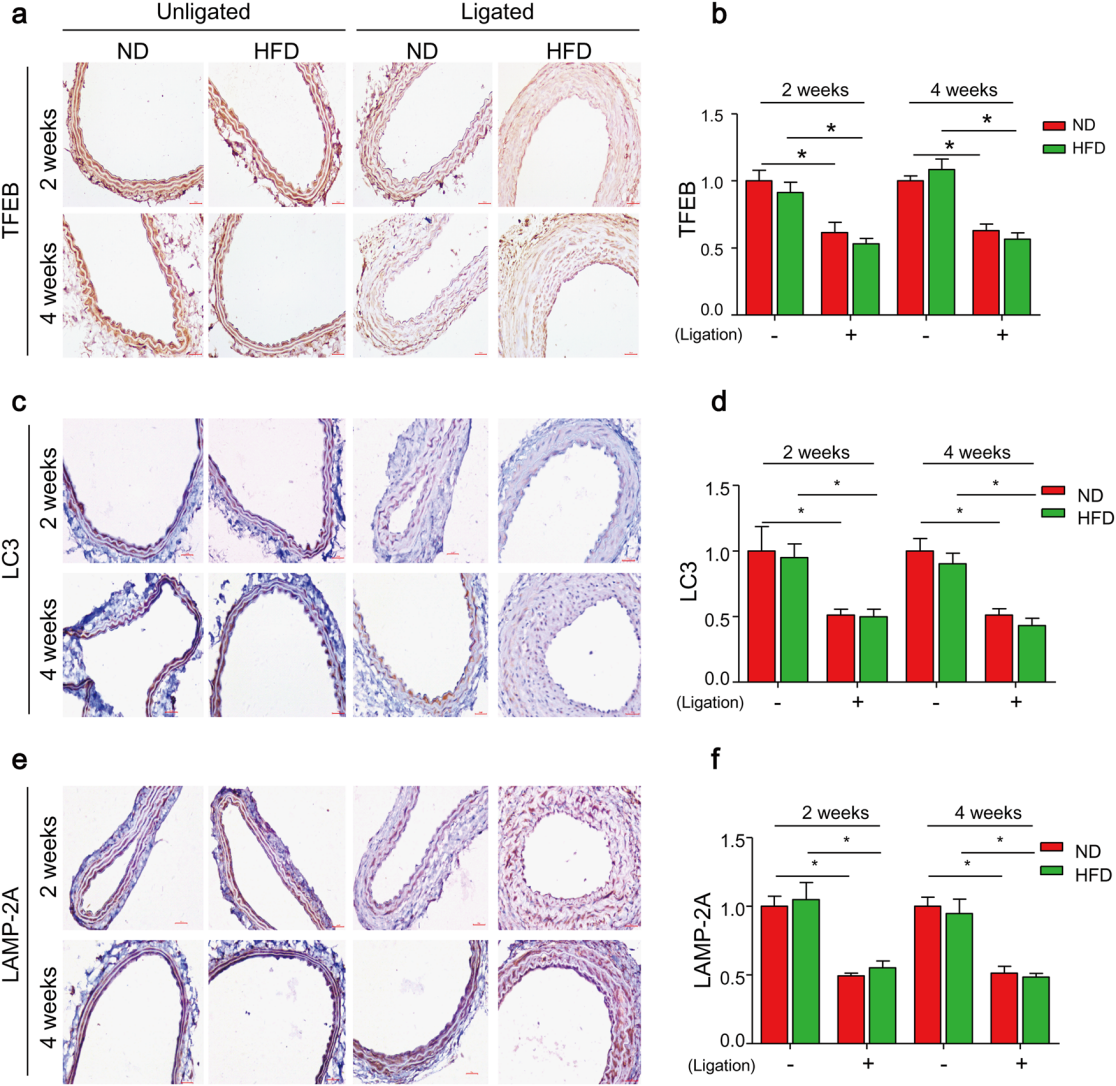
TFEB-mediated autophagy signaling is defective in arterial media of partial ligated carotid arteries (PLCAs). The left carotid arteries of mice were partially ligated (ligation) and mice were fed ND or HFD for 2–4 weeks after ligation, the right carotid arteries (unligated) of mice were served as control. **a**, **b** Representative IHC analysis of TFEB expression (brown color) in cross sections of carotid arteries. **c**, **d** Representative IHC analysis of LC3 expression (brown color) in cross sections of carotid arteries. **e**, **f** Representative IHC analysis of LAMP-2A expression (brown color) in cross sections of carotid arteries. Scale bar = 100 μm *P < 0.05 (*n* = 6–7)

We next determine whether TFEB signaling is impaired in the vasculature in a mouse model of vascular injury. In this model, the left carotid arteries of mice were partially ligated to induce local fluid flow reduction, which was shown to induce endothelial dysfunction and SMC dedifferenation in these partial ligated carotid arteries (PLCAs)^51,52^. In addition, mice were fed HFD to induce more severe vascular injury with neointima development^53^. We then examined the expression of TFEB and molecular markers for TFEB-autophagy signaling pathway by immunohistochemistry. TFEB expression was markedly decreased in the media of PLCAs in either ND- or HFD-fed mice at 2 and 4 weeks when compared with their unligated controls (Fig. 5a, b). Similarly, the expression of LC3 and LAMP-2A were also decreased in the media of PLCAs in either ND- or HFD-fed mice **(**Fig. 5c–f**)**. Together, these data suggest that TFEB-mediated autophagy signaling pathway is impaired in the arterial media of PLCAs, which is due to decreased fluid flow by partial ligation but not depends on HFD treatment.

### TFEB downregulation correlates with increased SMC dedifferentiation and enhanced HFD-induced neointima formation in PLCAs

**Fig. 6:**
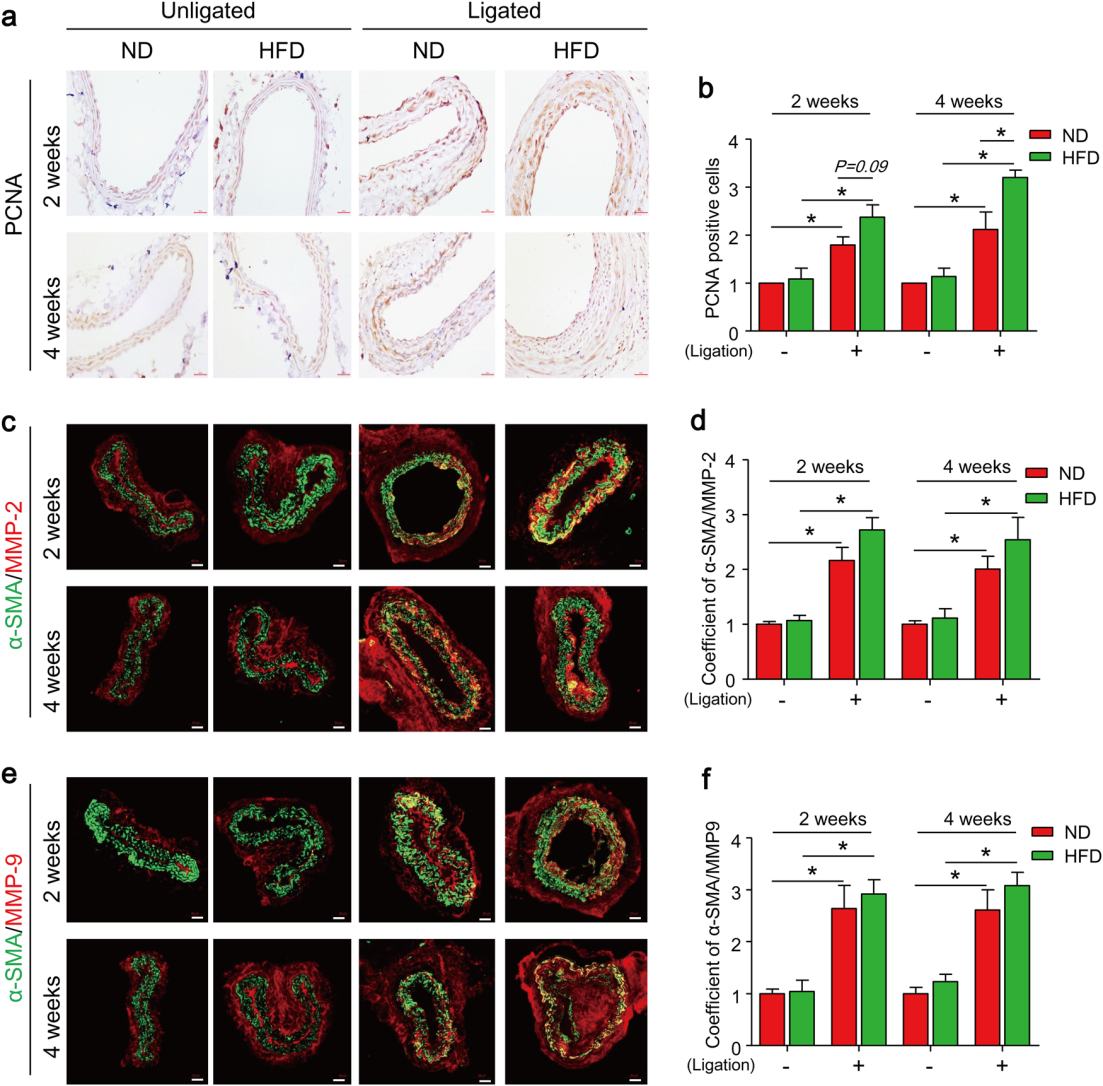
TFEB downregulation correlates with enhanced proliferation and migration of SMCs in PLCAs. The left carotid arteries of mice were partially ligated (ligation) and mice were fed ND or HFD for 2–4 weeks after ligation, the right carotid arteries (unligated) of mice were served as control. **a**, **b** Representative IHC analysis of PCNA expression (brown color) in cross sections of carotid arteries. **c**, **d** Representative immunofluorescence analysis of expression of MMP2 (red) and α-SMA (green) and their overlay (yellow) in cross sections of carotid arteries. **e**, **f** Representative immunofluorescence analysis of expression of MMP9 (red) and α-SMA (green) and their overlay (yellow) in cross sections of carotid arteries. Scale bar = 100 μm. *P < 0.05 (*n* = 6–7)

The abnormal proliferative and migrative potential of arterial SMCs into the intima layer are prominent features of the growth of neointima formation in vascular remodeling. We next determined whether TFEB expression is correlated with changes in proliferation and migration in vivo. Mice fed ND displayed increased proliferative potential in response to reduced fluid flow in PLCAs as measured by increased PCNA (proliferating cell nuclear antigen) staining (ligated vs. unligated), and these responses were enhanced by HFD treatment (Fig. 6a, b). ND fed mice also had higher expression of MMP2 and MMP9 in PLCAs compared with unligated arteries (Fig. 6c-f). However, HFD did not further enhanced MMP2 and MMP9 expression in PLCAs suggesting that these enzymes may be maximally activated by ligation alone. Meanwhile, HFD can significantly increase plasma cholesterol level (TC and LDL) and steatosis (Supplementary Fig. S4).

**Fig. 7:**
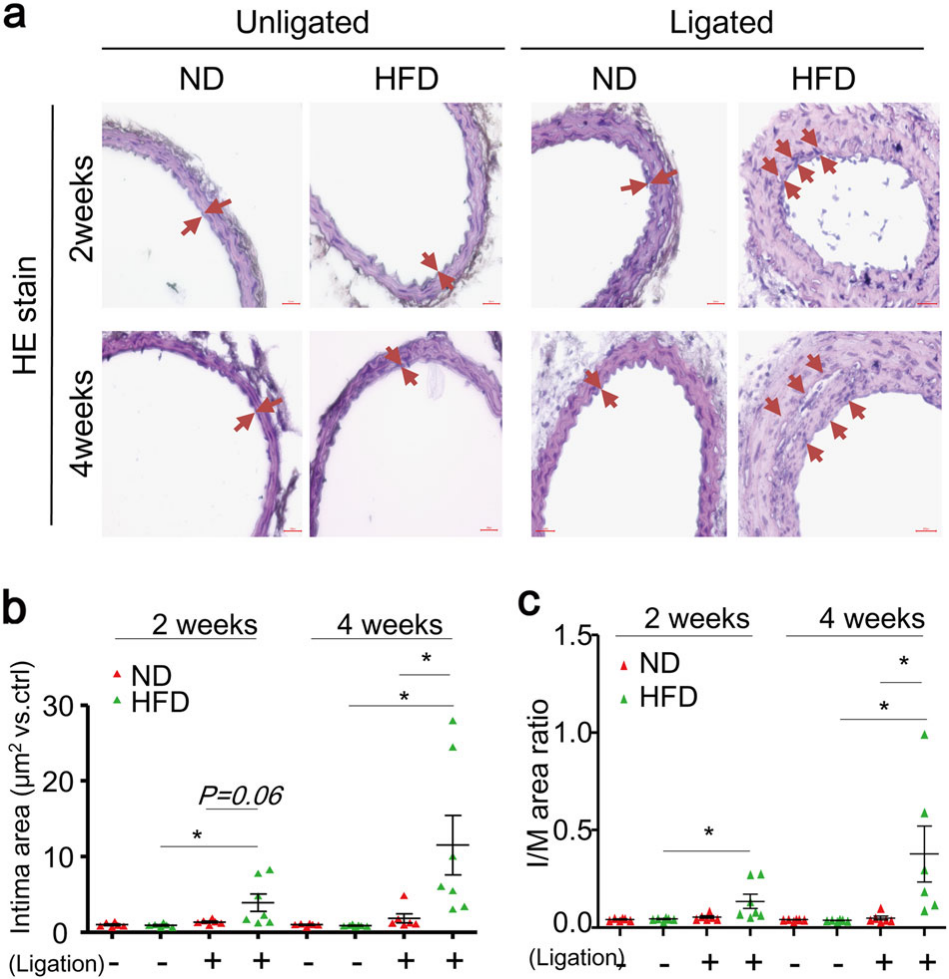
TFEB downregulation correlates with aggravated HFD-induced neointima formation in PLCAs. The left carotid arteries of mice were partially ligated (ligation) and mice were fed ND or HFD for 2–4 weeks after ligation, the right carotid arteries (unligated) of mice were served as control. **a** Representative H&E staining show the neointima formation in carotid arteries. Intima was indicated as regions between arrows. **b** Summarized data show the quantification of intima area in the lumen of carotid arteries. **c** Summarized data show the quantification of intima-over-media ratio of carotid arteries. Scale bar = 100 μm. *P < 0.05 (*n* = 6–7)

We next examined the effects of ligation (fluid flow reduction) and/or HFD treatment on neointima formation by analyzing intima area and intima-over-media ratio. As shown in Fig. 7a–c, neither ligation alone (ND + ligation) nor HFD alone (HFD + unligated) could trigger neointimal formation. In contrast, HFD treatment plus ligation (HFD + ligation) for either 2 or 4 weeks induced neointima formation (Fig. 7a–c).

### Trehalose inhibits SMC phenotypic modulation in vivo and prevents HFD-induced neointima in PLCAs

**Fig. 8:**
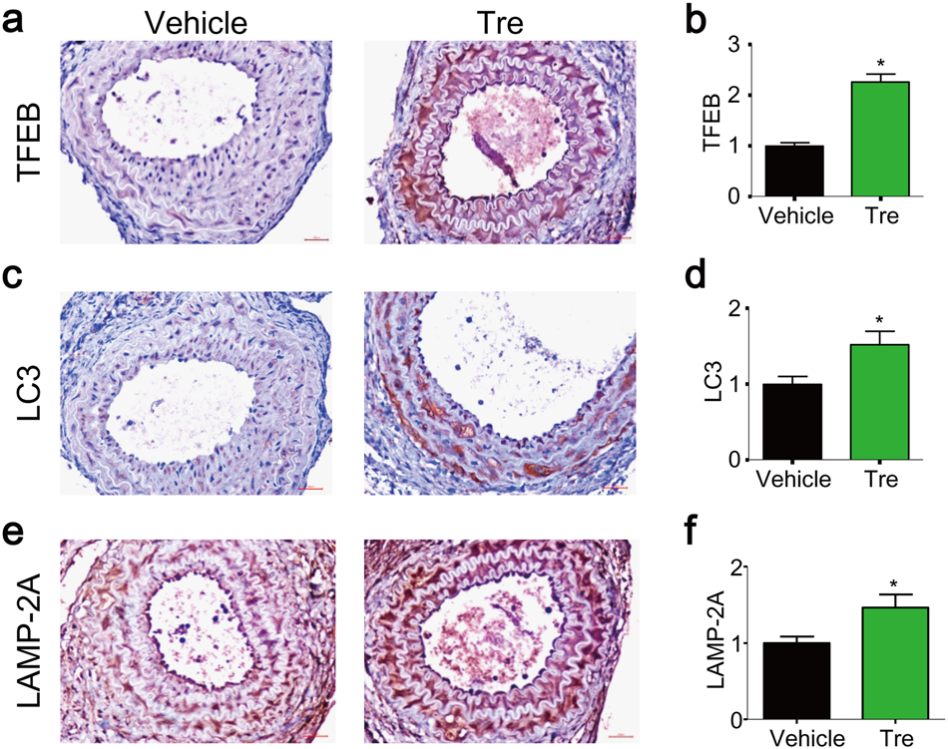
Trehalose enhances TFEB-mediated autophagy signaling in PLCAs. The left carotid arteries of mice were partially ligated (ligation), and after ligation mice were fed HFD and treated with vehicle (PBS) or trehalose (i.p. 2 g/kg, every 2 days) for 4 weeks. **a**, **b** Representative IHC analysis of TFEB expression (brown color) in cross sections of PLCAs. **c**, **d** Representative IHC analysis of LC3 expression (brown color) in cross sections of PLCAs. **e**, **f** Representative IHC analysis of LAMP-2A expression (brown color) in cross sections of PLCAs. Scale bar = 100 μm. *P < 0.05 (*n* = 7–8)

**Fig. 9:**
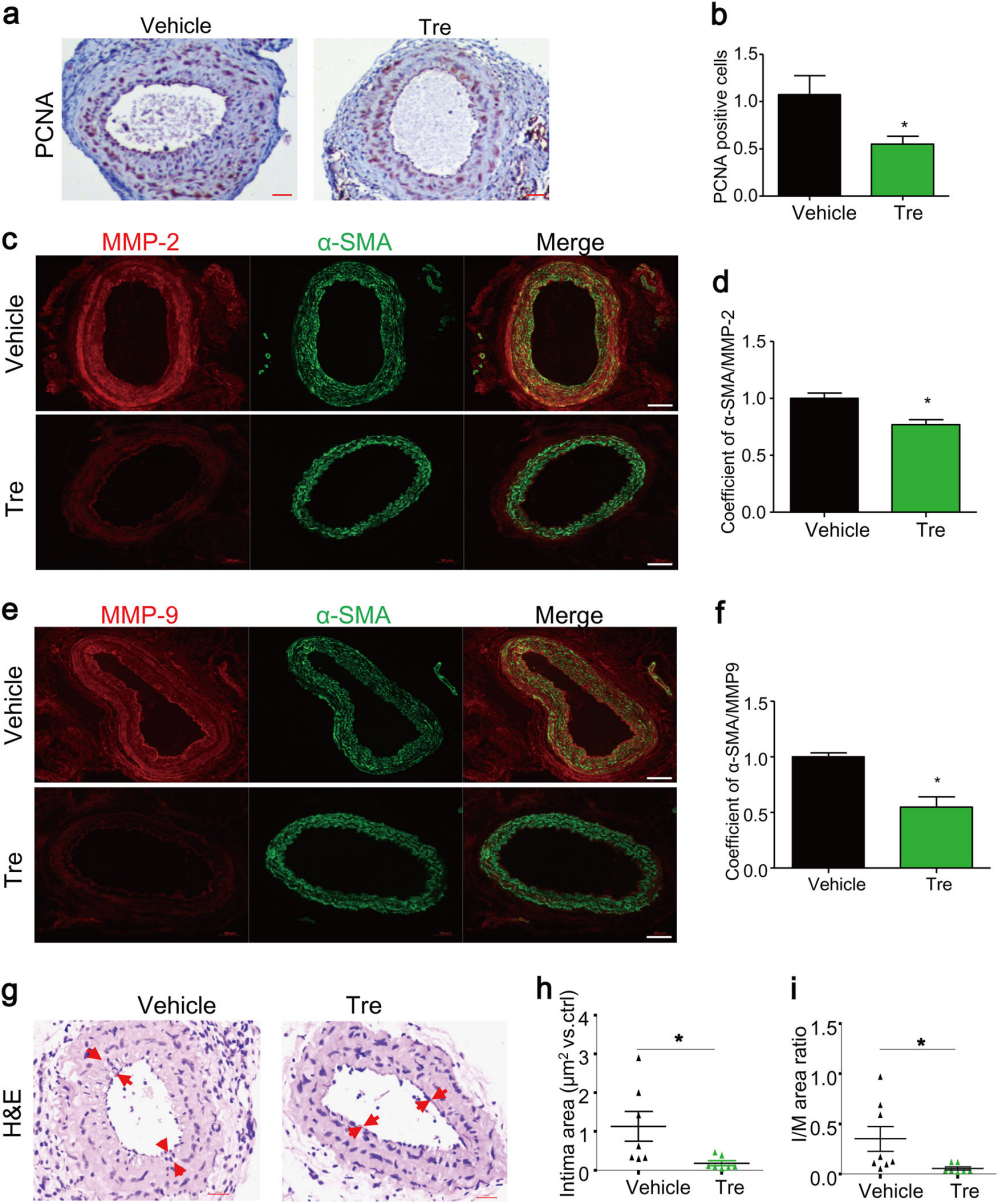
Trehalose attenuates proliferation and migration of SMCs and prevents HFD-induced neointima formation in PLCAs. The left carotid arteries of mice were partially ligated (ligation), and after ligation mice were fed HFD and treated with vehicle (PBS) or trehalose (i.p. 2 g/kg, every 2 days) for 4 weeks. **a**, **b** Representative IHC analysis of PCNA expression (brown color) in cross sections of PLCAs. **c**, **d** Representative immunofluorescence analysis of expression of MMP2 (red) and α-SMA (green) and their overlay (yellow) in cross sections of PLCAs. **e**, **f** Representative immunofluorescence analysis of expression of MMP9 (red) and α-SMA (green) and their overlay (yellow) in cross sections of PLCAs. **g**–**i** Representative H&E staining and quantification of intima area and intima-over-media ratio of cross sections of PLCAs. Scale bar = 100 μm. *P < 0.05 (*n* = 7–8)

Given the potent anti-proliferative and anti-migratory effects of trehalose on cultured SMCs, we examined whether or not trehalose could enhance TFEB signaling, attenuate SMC activation, and thereby prevent HFD-induced neointima formation in PLCAs. We found that trehalose treatment increased the expression levels of TFEB (Fig. 8a, b), LC3 (Fig. 8c, d), and LAMP-2A (Fig. 8e, f) in the media of PLCAs of mice fed HFD. These data suggest that trehalose reactivates TFEB-mediated autophagy signaling pathway in PLCAs. Trehalose also significantly inhibited the proliferation and migration of SMCs in these PLCAs as shown by decreased expression levels of PCNA **(**Fig. 9a, b**)**, MMP-2 **(**Fig. 9c, d**)**, and MMP-9 **(**Fig. 9e, f). Consistently, trehalose remarkably attenuated the HFD-induced neointima formation in PLCAs **(**Fig. 9g–i**)**. Trehalose was recently reported to have anti-obesity effects^54,55^. However, in our experimental settings, HFD treatment up to 4 weeks did not significantly increase body weight, and trehalose did not affect body weight, plasma cholesterol levels (TC and LDL), and steatosis in mice fed HFD (Supplementary Fig. S5). These data suggest that trehalose may not affect the early onsets of obesity associated with HFD. Taken together, our data indicate that activation of TFEB by trehalose can effectively attenuate proliferation and migration of SMCs and prevent HFD-induced intimal hyperplasia and neointima formation in PLCAs.

## Discussion

The present study indicated that activation of TFEB enhances autophagy signaling in SMCs in vitro and in arterial media in vivo. In cultured SMCs, activation of TFEB by trehalose or its ectopic expression inhibited proliferation and migration. Our animal experiments demonstrated that TFEB-mediated autophagy signaling was suppressed in PLCAs, which correlates with pathological vascular remodeling as characterized by enhanced synthetic phenotype switching and aggravated neointima formation by HFD. Trehalose effectively reactivated TFEB-autophagy signaling and blocked this pathological vascular remodeling. These results suggest that suppression of TFEB-mediated autophagy signaling may be an important initiating mechanism to promote SMC dedifferentiation, leading to enhanced synthetic phenotype switching and accelerated vascular remodeling under metabolic stress associated HFD.

Serum stimulation promotes SMCs dedifferentiation leading to their proliferation and migration. In this study, we demonstrated that serum stimulation suppressed TFEB activity in SMCs compared with that in SMCs under serum reduced condition, a phenomenon that was reported in other cell types due to increased contents of nutrients and growth factors^56^. Moreover, we observed that trehalose increased intracellular levels of autophagosome marker LC3 and autophagic substrate p62/SQSTM1, and nuclear translocation of TFEB in cultured SMCs with serum stimulation. Arresting autophagic flux could further enhance trehalose-induced LC3 expression in SMCs. More importantly, TFEB gene silencing could inhibit trehalose-induced changes in LC3 and p62/SQSTM1 (Fig. 4a). Undoubtedly, the potentiating effect of trehalose is attributed to TFEB-mediated transcriptional upregulation of TFEB itself and the autophagy genes LC3 and p62/SQSTM1 but not due to inhibition of autophagic flux. In addition, trehalose also resulted in upregulation of lysosome gene LAMP-2A. The functional role of TFEB in autophagy signaling was also confirmed in SMCs using ectopic expression of TFEB. Together, the present study provided the first evidence that TFEB-autophagy signaling can be reactivated or potentiated in SMCs.

The present study is the first to address the functional role of TFEB-mediated autophagy signaling in SMC biology and pathology. Recent studies revealed a direct relationship between autophagy signaling and SMC proliferation. Atherogenic stimulation of coronary arterial SMCs with 7-ketocholesterol elicits autophagy as well as proliferation^7,57^. Such 7-ketocholesterol-induced SMC proliferation was dramatically enhanced by gene deletion of a lysosomal acid sphingomyelinase or an Nicotinic acid adenine dinucleotide phosphate (NAADP) metabolizing enzyme CD38 that regulates lysosome function involved in autophagosome fusion with a lysosome to form autophagolysosome^7,57^. Moreover, free fatty acids including oleic acid and palmitic acid enhanced autophagy flux that suppressed proliferation of aortic SMCs^58^. In line with these findings, the present study demonstrated that activation of TFEB by trehalose or its ectopic expression markedly inhibited proliferation of SMCs under serum stimulation. Progression through the cell cycle phases is regulated by cyclin-dependent kinases and their regulatory cyclin subunits. TFEB activation may inhibit cells entering G1 phase as both trehalose and TFEB overexpression downregulated cyclin D1 and CDK4, two regulators for G1 phase. Existing evidences demonstrated augmented autophagy by rapamycin inhibits SMC migration^18,19^. Consistently, TFEB activation was also manifested to inhibit SMC migration induced by serum, and this inhibition was associated with reorganized F-actin and a reduction of MMP-2 expression. The detailed mechanisms of autophagy regulating SMC proliferation and migration remain unclear and deserve further elucidation.

In our animal experiments, we characterized the TFEB-autophagy signaling pathway in arterial wall and determined the effects of fluid flow reduction by partial carotid ligation or metabolic stress by HFD. Our data demonstrated that partial carotid ligation independently suppressed the TFEB-autophagy signaling as early as 2 weeks post ligation, whereas TFEB pathway was not affected by HFD treatment alone or further suppressed by combination of ligation with HFD (Fig. 5). The present study did not attempt to further investigate the molecular mechanisms linking fluid flow reduction and TFEB suppression in the arterial wall. Recent studies showed that TFEB activity is inversely related to mTORC1 activation, which increases TFEB phosphorylation and promotes its degradation in the cytoplasma^38,59,60^. Normal endothelial cells respond to physiological fluid flow by releasing a variety of microRNAs including miR-100, which suppresses mTORC1 in arterial SMCs^61,62^. In case of reduced fluid flow, endothelial release of miR-100 is limited that may cause mTORC1 activation and consequent suppression of TFEB activity in arterial SMCs. Future studies will investigate these molecular mechanisms in vitro and in vivo.

Our analysis of proliferation and migration related molecular markers in PLCAs further revealed that the TFEB suppression by fluid flow reduction was correlated with enhanced proliferative and migratory potential in vivo (Fig. 6), but not associated with neointima development (Fig. 7). Our finding is in accordance with previous reports that partial carotid ligation can reduce fluid flow, which in turn induces endothelial dysfunction and SMC dedifferentiation, but is insufficient to trigger neointimal lesions^51,63^. It should be noted that this “partial” carotid ligation model is different from a commonly used “complete” carotid ligation model, in which fluid flow cessation induces more severe endothelium injury (mostly denudation) and neointima formation^64^. Despite of the absence of neointima in unligated carotid arteries of mice fed HFD, our data demonstrated that HFD treatment can increase PCNA-positive cells and induce neointima formation in PLCAs (Figs. 6a and 7). Together, these data support the view that TFEB suppression (e.g. by fluid flow reduction) plays a role in reprogramming SMCs with dedifferentiated status such as proliferative and migratory potential in vivo, which sensitizes or accelerates HFD-induced intimal hyperplasia leading to neointima development.

To further explore the role of TFEB in SMC phenotypic plasticity in vivo, we treated mice with trehalose and observed changes in SMC dedifferentiation markers and neointimal lesions in PLCAs under HFD condition. Trehalose effectively enhanced TFEB-autophagy signaling in the arterial media of PLCAs as shown by increased expression of TFEB and its downstream genes LC3 and LAMP-2A (Fig. 8). Moreover, TFEB activation in the arterial media by trehalose inhibited PCNA-positive cells, MMP-2 and MMP-9 expression, and neointima development in PLCAs (Fig. 9). Our data suggest that reactivation of TFEB signaling could reverse the SMC dedifferentiation in the arterial wall that impedes the neointima development by HFD. Vascular SMC proliferation and migration are essential to the development of neointima, a process involves a large number of events in response to injury leading to SMC dedifferentiation. It has been well documented that endothelial dysfunction or macrophage activation can crosstalk with SMC dedifferentiation in the arterial walls through releasing various mediators including inflammatory cytokines, growth factors, reactive substances, and miRNAs. Indeed, previous studies demonstrated that trehalose enhanced TFEB-dependent autophagy signaling in macrophages that inhibits inflammation contributing to its protective effects on atherosclerosis^42,43^. Endothelial specific overexpression of TFEB suppresses endothelial inflammation and attenuate atherosclerosis in mice^44^. In humans, oral trehalose supplementation improves endothelial function in resistance artery^65^. Therefore, these previous reports imply that trehalose may also prevent SMC dedifferentiation indirectly through its protective effects on endothelial dysfunction or macrophage activation. The data from the present study did not exclude these possibilities. A future study with SMC specific TFEB overexpression in the arterial media may more precisely address this concern. Nonetheless, in our experimental settings, the inhibitory effects of trehalose on SMC dedifferentiation and HFD-induced neointima formation may be, at least in part, due to reactivation of TFEB-autophagy signaling in SMCs.

In summary, our results showed that TFEB plays a critical role in maintaining the quiescent status of arterial SMCs in vitro and in vivo. Downregulation of TFEB-mediated autophagy signaling axis correlates with medial dedifferentiation in fluid flow reduced arteries. TFEB activator trehalose reverses such phenotypic switching and prevents HFD-induced intimal hyperplasia and neointima formation. Our findings provide novel insights into therapeutic effects of trehalose on vascular dysfunction associated with HFD through activation of TFEB signaling in arterial SMCs.

## Materials and methods

### Antibodies and reagents

Primary antibodies: LC3 (CST 12741S), β-actin (CST 3700S), PCNA (CST 13110), p62/SQSTM1 (Abcam ab109012), LAMP-2A (ab18528), Ki67 (Abcam ab16667), MMP-2 (Abcam ab92536), α-smooth muscle actin (α-SMA) (Abcam ab5694), cyclin D1 (BD 554181), CDK4 (BD 559677), TFEB (Bethyl Laboratories A303-673A), and MMP-9 (Santa Cruz sc-13520), DDK-FLAG (Origene TA50011-100). Secondary antibody for western blot: IRDye® 800CW donkey anti-Mouse IgG (H + L) (LICOR 926-32212); IRDye® 800CW donkey anti-rabbit IgG (H + L) (LICOR 926–32213); donkey anti-mouse IgG (H + L) secondary antibody, HRP (Thermo Fisher A16011); stabilized peroxidase conjugated goat anti-rabbit (H + L) (invitrogen32460); goat anti-rat IgG-HRP (Thermo Fisher 629520). Secondary antibody for immunofluorescence: donkey anti-mouse IgG (H + L) secondary antibody, Alexa Fluor® 488 conjugate (Thermo Fisher A-21202); donkey anti-rabbit IgG (H + L) secondary antibody, Alexa Fluor® 488 conjugate (Thermo Fisher A21206); donkey anti-mouse IgG (H + L) secondary antibody, Alexa Fluor® 555 conjugate (Thermo Fisher A-31570); donkey anti-rabbit IgG (H + L) secondary antibody, Alexa Fluor® 555 conjugate (Thermo Fisher A-31572). Secondary antibody for IHC: biotinylated anti-rabbit igG(H + L) (vector laboratories, BA-1000) or goat anti-mouse (vector laboratories, BA-9200).

Reagents: Aurum Total RNA Mini Kits (Bio-Rad, Cat. #732-6820, USA), iScript Reverse Transcription Supermix for RT-qPCR (Bio-Rad, Cat. #1708841, USA), iTaq Universal SYBR Green supermix (Bio-Rad, Cat. #1725121, USA), Rhodamine Phalloidin (Fisher R415), Trehalose (Fisher 62-562-550GM), and Bafilomycin (Sigma, B1793). HRP, DAB peroxidase substrate (vector laboratories SK-4100), HE-stain kit (Beyotime C0105, China), total cholesterol (TC) assay kit (NanJING Jiancheng Bioengineering institute A111-1, China), low-density lipoprotein cholesterol (LDL-C) assay kit (NanJing Jiancheng Bioengineering institute A113-1, China).

### Mice

All experimental protocols were reviewed and approved by the Animal Care Committee of University of Houston and Guangzhou University of Chinese Medicine. C57BL/6J male mice were used in all experiments. All animals were provided standard rodent chow, water ad libitum and 12 h dark/light cycles in a temperature-controlled room.

### Primary culture of arterial SMCs from mice

SMCs were isolated from mice as previously described^27,66^. Six-week-old male C57BL/6J mice were used in the present study. In brief, mice were deeply anesthetized with intraperitoneal injection of pentobarbital sodium (25 mg/kg). The heart was excised with an intact aortic arch and immersed in a petri dish filled with ice-cold Krebs–Henseleit solution. A 25-gauge needle filled with Hanks’ buffered saline solution was inserted into the aortic lumen opening while the whole heart remained in the ice-cold buffer solution. The opening of the needle was inserted deep into the heart close to the aortic valve. The needle was tied in place with the needle tip as close to the base of the heart as possible. The infusion pump was started with a 20-ml syringe containing warm HBSS through an intravenous extension set at a rate of 0.1 ml/min for 15 min. HBSS was replaced with warm enzyme solution (1 mg/ml collagenase type 1, 0.5 mg/ml soybean trypsin inhibitor, 3 % bovine serum albumin, and 2% antibiotic), which was flushed through the heart at a rate of 0.1 ml/min. Perfusion fluid was collected at 30, 60, and 90-min intervals. At 90 min, the heart was cut with scissors, and the apex was opened to flush out the cells that collected inside the ventricle. The fluid was centrifuged at 1000 rpm for 10 min, the cell-rich pellets were mixed with the Advanced Dulbecco’s modified Eagle’s medium with 10% fetal bovine serum, 10% mouse serum, and 2% antibiotics. The isolated cells were plated on 2% gelatin-coated six-well plates and incubated in 5% CO_2_ at 37 °C. These isolated cells were considered as SMCs originated mainly from coronary arteries by positive staining with α-SMA antibodies and the SMCs morphology. The medium was replaced 3 days after cell isolation and then once or twice each week until the cells grew to confluence. All studies were performed with cells of passage of 5–8.

### Partial carotid ligation surgery

Partial carotid ligation was performed as previously reported^51,63^. In brief, C57BL/6J male mice at 8–10 week of age were anesthetized by 2% isoflurane on the 37 °C heating pad. Epilated area was disinfected with 70% ethyl alcohol and betadine. A cervical longitudinal median incision (~5 mm) was made. Left carotid artery (LCA) was gently exposed by blunt dissection. Three of four branches of LCA (left external carotid, internal carotid, and occipital artery) were ligated with 6–0 silk suture. The superior thyroid artery was left intact to provide the blood flow. After surgery, the incision was closed with 4–0 absorbable suture. A single subcutaneous injection of buprenorphine (0.1 mg/kg) was given after mice recover from surgery for additional pain relief. Mice were randomly assigned and fed regular chow (normal diet, ND) or a HFD containing 35% kcal fat, 1.25% cholesterol, and 0.5% sodium cholate (Trophic Animal Feed High-tech Co. Ltd) for 2 or 4 weeks. In the trehalose treatment and vehicle control groups, all mice were randomly assigned and received partial carotid ligation surgery, and fed with HFD for 4 weeks. After the surgery, the mice in trehalose group or vehicle control group were also injected intraperitoneally with trehalose (2 g/kg, every 2 days) or vehicle (PBS) as described^42^. At the end of experiments, mice were sacrificed by cervical dislocation after administration of anesthesia. Blood samples were collected; LCAs and right carotid arteries (unligated controls) were then harvested for immunohistochemistry and fluorescence analyses. Frozen tissue samples were embedded in tissue processing medium (tissue-tek O.C.T), and immediately frozen in liquid nitrogen, and then transfer to −80 °C refrigerator for storage. Paraffin-embedded samples were harvested and kept in 4% buffered paraformaldehyde in 0.1 M sodium phosphate buffer, pH 7.4. After dehydration with the different concentration ethanol and xylene, samples were embedded in paraffin wax. The thickness of sections is 4.5 μm.

### Immunoblotting

Cells were lysed in Laemmli sample buffer (Bio-Rad, 161–0737) containing β-mercaptoethanol (Sigma Aldrich, M3148) to prepare whole cell lysates. Cytosolic and nuclear protein were extracted using NE-PER Nuclear and Cytoplasmic Extraction Reagents (Thermo Fisher 78833). Protein samples were boiled for 10 min at 95 °C, then placed in precooled water ultrasonic bath. Twenty micrograms of total proteins was separated by 8–12% sodium dodecyl sulfate-polyacrylamide gel electrophoresis. The proteins of these samples were then electrophoretically transferred at 35 V at 4 °C overnight onto a PVDF membrane (Bio-Rad, USA). The membrane was blocked with 5% nonfat milk in Tris-buffered saline. After washing, the membrane was probed with primary antibody as indicated according to the manufacturer’s instructions. After washing, the membranes were then incubated with corresponding secondary antibodies and bands were visualized and analyzed by the LI-COR^®^ Odyssey Fc System^27^.

### Plasmid transfection

SMCs were transfected with TFEB cDNA plasmids to overexpress TFEB gene in SMCs. In brief, 10^5^ SMCs were cultured in six-well plate till they reached ~70% confluency and then cells were transfected with 3 µg scramble cDNA plasmid or TFEB-DDK-FLAG cDNA plasmid (Origene, MR223016) in LyoVec^TM^ reagents (Invivogen, lyec-1) for 24 h according to the manufacturer’s instructions. The transfection efficiency was examined by western blot analysis of DDK-FLAG and the downstream proteins of TFEB pathway.

### Quantitative real-time PCR

Total RNA was isolated using the Aurum Total RNA Mini Kits (Bio-Rad, Cat. #732-6820, USA) according to the manufacturer’s instructions. cDNA was generated from the RNA using iScript Reverse Transcription Supermix for RT-qPCR (Bio-Rad, Cat. #1708841, USA). Real-Time PCR was performed using the iTaq Universal SYBR Green supermix (Bio-Rad, Cat. #1725121, USA) on the Bio-Rad CFX Connect Real-Time System using the following primers: TFEB forward primer:5′-CAGCAGGTGGTGAAGCAAGAGT (22mer)-3′; TFEB reverse primer:5′-TCCAGGTGATGGAACGGAGACT (22mer)-3′. LC3 forward primer:5′-CGTCCTGGACAAGACCAAGT-3′; LC3 reverse primer:5′-ATTGCTGTCCCGAATGTCTC-3′. p62/SQSTM1 forward primer: 5′-AGGGAACACAGCAAGCT-3′; p62/SQSTM1 reverse primer:5′-GCCAAAGTGTCCATGTTTCA-3′. LAMP-2A forward primer:5′- CCAAATTGGGATCCTAACCTAA-3′; LAMP-2A reverse primer:5′-TGGTCAAGCAGTGTTTATTAATTCC-3′. β-actin forward primer:5′- TCGCTGCGCTGGTCGTC-3′; β-actin reverse primer:5′- GGCCTCGTCACCCACATAGGA-3′. The cycle threshold values were converted to relative gene expression levels using the 2^−ΔΔCt^ method. The data were normalized to that of internal control β-actin.

### Immunofluorescence staining

For cultured cells, ~1 × 10^4^ SMCs were seeded on gelatin-coated coverslips in 24-well plate. Cells were stimulated as indicated, washed with PBS, and then fixed with 4% paraformaldehyde for 15 min at room temperature. After fixation, cells were washed twice with PBST (0.05% Tween20 in PBS) and permeabilized with 0.3% Triton X-100 in PBST for 15 min. Nonspecific sites were blocked with 5% BSA in PBS at room temperature for 1 h, and then cells were incubated with indicated primary antibodies at 4 °C overnight. For frozen tissue sections, samples were fixed in precold acetone in −20 °C for 15 min, washed, and permeabilized as above. The tissue sections were blocked with 10% donkey serum in PBS for 30 min followed by incubation overnight at 4 °C with indicated primary antibodies. Sections were then incubated with corresponding secondary antibodies conjugated with Alexa Fluor 488 or Alexa Fluor 555 for 1 h at room temperature. The cell nucleus was stained with DAPI for 15 min at room temperature and mounted with anti-fluorescence quenching agent. For phalloidin staining of F-actin in cultured cells, fixed and permeabilized cells were incubated with Alexa-Fluor 568-conjugated phalloidin (1:50) for 30 min, washed with PBS, and mounted with anti-fluorescence quenching agent. Imaging was performed using ZEISS LSM800 or Olympus IX73 imaging system. The Pearson's correlation for co-localization efficiency and mean fluorescence density were analyzed using Image-Pro Plus 6.0 software as described previously^63^.

### Immumohistochemical staining

For paraffin-embedded tissue sections, after deparaffination by xylene and ethanol, peroxidase treatment in methanol with 0.5% hydrogen peroxide was followed by heat-assisted antigen retrieval in 0.01 M sodium citrate buffer (pH 6.0). After washed and permeabilized cells as immunofluorescence staining, blocked the nonspecific sites by 5% goat serum in PBS for 30 min followed by incubation with primary antibody at 4 °C overnight. Corresponding secondary antibody biotinylated anti-rabbit igG(H + L) (vector laboratories) or goat anti-mouse (vector laboratories) was added to sections for 30 min at room temperature. Then incubated with streptavidin-HRP for 30 min at room temperature. After wash, added DAB (vector laboratories) to each slide (1–2 min). When you see the background turn brown, washed in water. Counter stained with heamatoxylin. After wash in ethanol and xylene, added Neutral gum to slide and add coverslips to each tissue section to save slides. Imaging was performed using Olympus IX73 imaging system. The mean optical density of protein expression level was detected using software Image-Pro Plus 6.0 to quantify the protein expression.

### Migration assay

Cell migration was assessed by a wound scratch assay. Briefly, 90% confluent SMCs were starved in low-serum media (0.1% FBS) overnight. Scratch wounds were created using a 2 mm wide pipette tip. Cells were cultured in full-serum medium (10% FBS) with indicated treatment. After 24 h, the scratched areas of cells were imaged by using Olympus IX73 imaging system. Average scratch area was quantified using Image-Pro Plus 6.0 software.

### Lentiviral transduction

TFEB shRNA lentiviral particles were from Santa Cruz (sc-38510-V) and control shRNA lentiviral particles were from Origene (TR30037). SMCs were infected with the lentiviral particle in the presence of polybrene (final concentration was 8 μg/ml). After 48 h transduction, puromycin (2 μg/ml) was added to the media for selection. Surviving cells were allowed to proliferate for another 24 h and were used for downstream analyses. The effect of gene silencing on TFEB expression was then analyzed by Immunoblot analysis (Supplementary Fig. S3).

### MMP activity assay

A fluorometric-based MMP Activity Assay Kit (Abcam, ab112146) was used to analyze total MMP activity according to the manufacture’s instruction. SMCs after lentiviral transduction and selection were cultured in 10% FBS medium in 96 well plate till confluency. Cells were then treated with or without 100 mM trehalose for 48 h. Fifty microliters of fluorescence resonance energy transfer peptide as a generic MMP activity indicator was added to each well and incubated for 30 min. Then the fluorescence intensity (arbitrary unit) was measured at excitation/emission of 485/520 nm by a microplate reader (BMG Labtech).

### Statistics analysis

Data are presented as mean ± standard error. All experiments were analyzed by the Student’s *t* test or one/two-way ANOVA with treatments as category factors, followed by a Bonferroni's multiple comparisons test if applicable. A Student’s*t* test was used to detect significant difference between two groups. The statistical analysis was performed by Graphpad Prism 6.0 software (GraphPad Software, USA). *P* < 0.05 was considered statistically significant.

## Supplementary information

Supplemental document
