# Supplementary material for: Activation of TFEB ameliorates dedifferentiation of arterial smooth muscle cells and neointima formation in mice with high-fat diet
Source: Cell Death Dis. 2019 Sep 12;10(9):676. doi: 10.1038/s41419-019-1931-4 (PMC6742653; doi:10.1038/s41419-019-1931-4)
Supplement: Supplementary file 1 — Supplemental document [file 41419_2019_1931_MOESM1_ESM.pdf]

Supplemental Information

Activation of TFEB ameliorates dedifferentiation of arterial smooth muscle cells and neointima formation in mice with high fat diet

Yun-Ting Wang[1,2], Xiang Li[2], Jiajie Chen[2], Bradley K. McConnell[2], Li Chen[3], Pin-Lan Li[4], Yang Chen[1], Yang Zhang[2]

[1]School of Pharmaceutical, Guangzhou University of Chinese Medicine, Guangzhou, China;

[2]Department of Pharmacological and Pharmaceutical Sciences, College of Pharmacy, University of Houston, Houston, USA

[3]Department of Biology and Biochemistry, University of Houston, Houston, USA

[4]Department of Pharmacology and Toxicology, School of Medicine, Virginia Commonwealth University, Richmond, USA

Correspondence to:

Yang Zhang, PhD, Department of Pharmacological & Pharmaceutical Sciences, College of Pharmacy, University of Houston, Houston, TX 77204-5056, Tel: 7137437710, Fax: 7137431259, Email: yzhan219@central.uh.edu or Yang Chen, PhD, School of Pharmaceutical, Guangzhou University of Chinese Medicine, Guangzhou 51000, China; ychen8@gzucm.edu.cn

Supplementary Figures S1

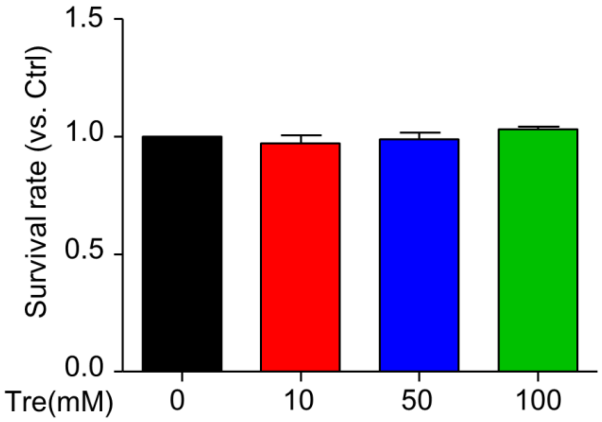

Figure S1. **Trehalose does not affect cell survival in SMCs.** SMCs were cultured in full-serum medium (10% FBS) with trehalose (0-100 mM) for 24 hours. The cytotoxicity of trehalose (0-100 mM) was analyzed by LDH (Lactate dehydrogenase) assay. LDH activity in the cell culture medium was determined by Pierce™ LDH Cytotoxicity Assay Kit (Thermo, Rockford, IL, USA) according to the manufacturer's protocol.

Supplementary Figure S2

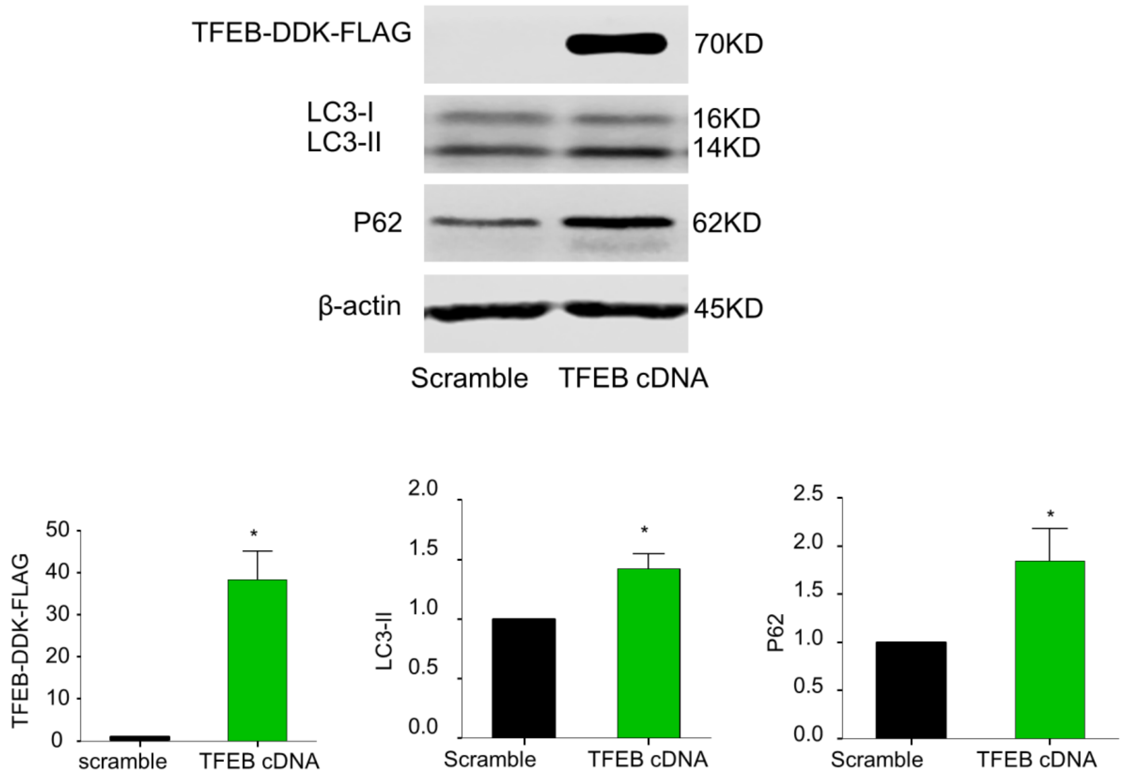

Figure S2. Ectopic expression of TFEB upregulates TFEB-autophagy signaling in SMCs. SMCs were transfected with scramble or TFEB cDNA plasmids (TFEB-DDK-FLAG plasmid, Origene, MR223016) for 24 hours as described in *Method*s. The transfection efficiency of TFEB was examined by Western blot analysis of TFEB-DDK-FLAG (anti-FLAG), LC3 and p62. *P< 0.05 (n = 4).

Supplementary Figure S3

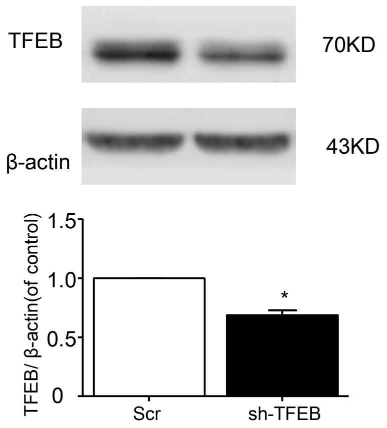

Figure S3. TFEB gene silencing down-regulates TFEB expression in SMCs. SMCs were transduced with scramble or TFEB shRNA lentiviral particles as described in *Method*s. The transfection efficiency of TFEB was examined by Western blot analysis of TFEB expression. *P< 0.05 (n = 4).

Supplementary Figure S4

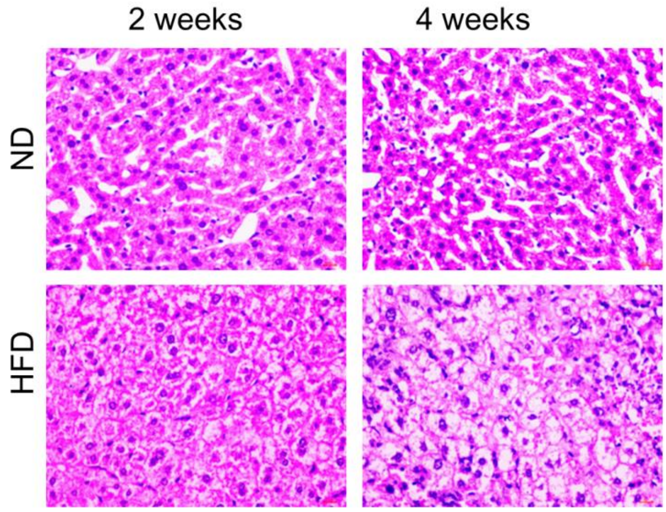

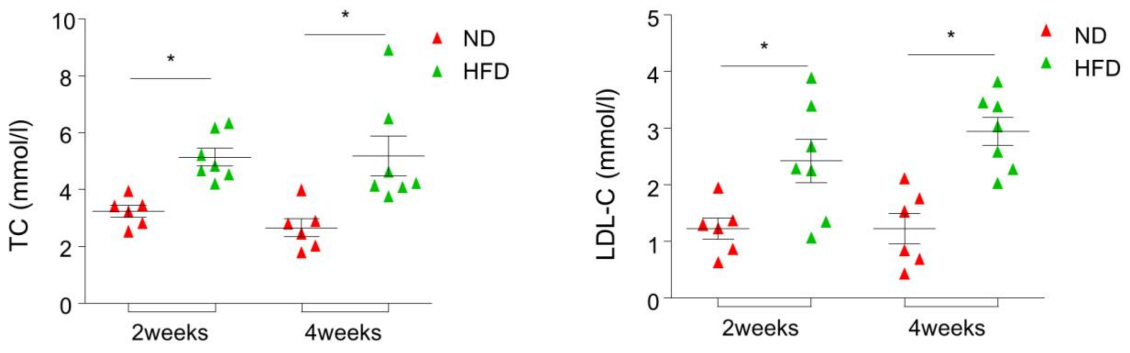

Figure S4. Effects of high fat diet (HFD) on plasma cholesterol and steatosis in mice. Mice were fed normal diet (ND) or HFD for 2 or 4 weeks. HFD induced steatosis (IHC images) and increased plasma total cholesterol (TC) and LDL-cholesterol (LDL-C). *P< 0.05 (n = 6-7).

Supplementary Figure S5

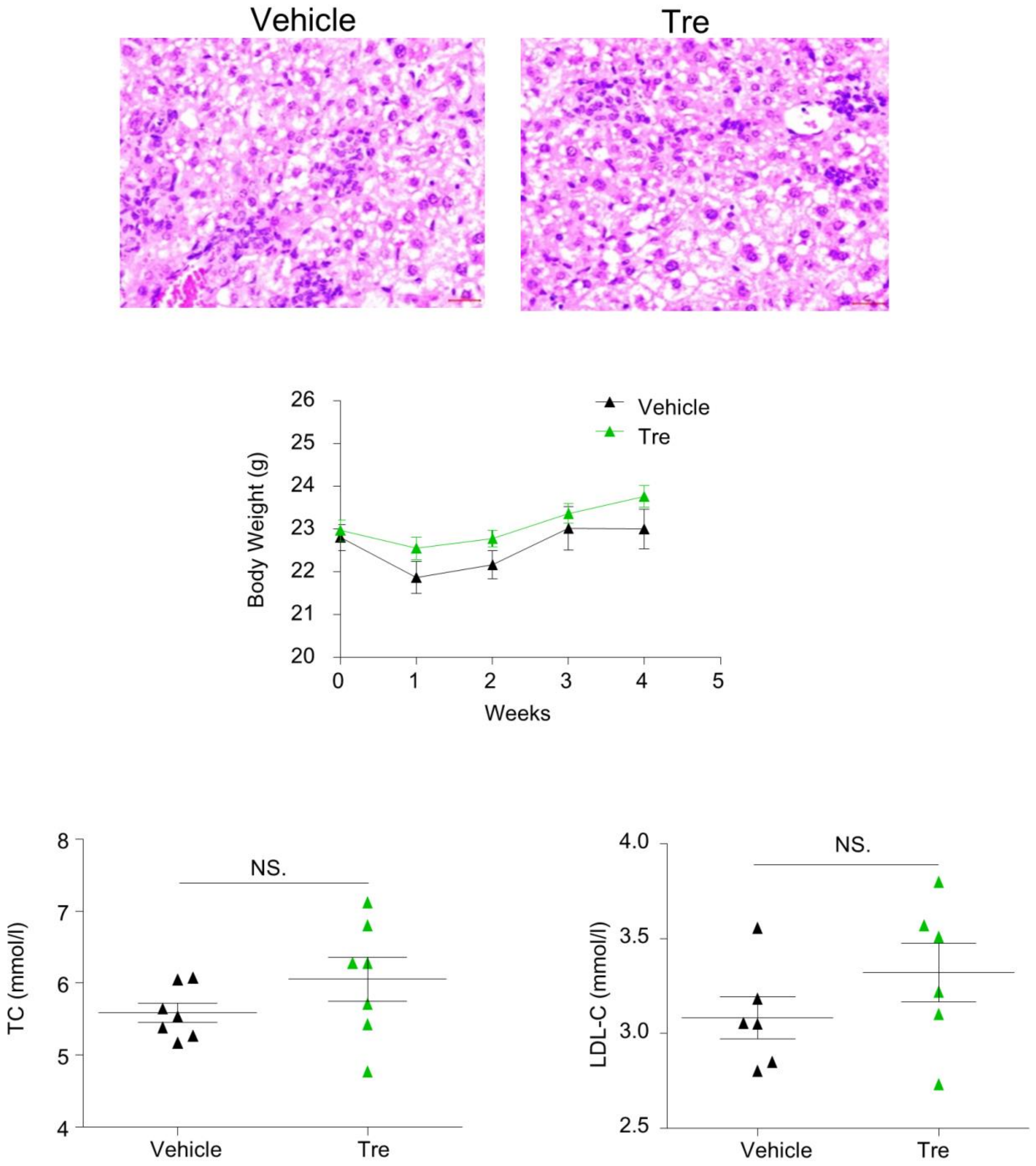

Figure S5. Effects of trehalose on HFD-induced steatosis and plasma cholesterol in mice. Mice were fed HFD and treated with vehicle (PBS) or trehalose (i.p. 2g/kg, every two days) for 4 weeks. Trehalose did not affect body weight, plasma cholesterol levels (TC and LDL), and steatosis (IHC data).
